# Supplementary material for: Splice-Junction-Based Mapping of Alternative Isoforms in the Human Proteome
Source: Cell Rep. Author manuscript; Available in PMC 2020 Jan 15. (PMC6961840; doi:10.1016/j.celrep.2019.11.026)
Supplement: 2 [file NIHMS1546469-supplement-2.zip › DF1/PXD010154/Ovary/Ovary_6_MAP4_MYHDDDLADLVFPSSATADTSIFAGQNDPLKDSYVPLELAK.pdf]

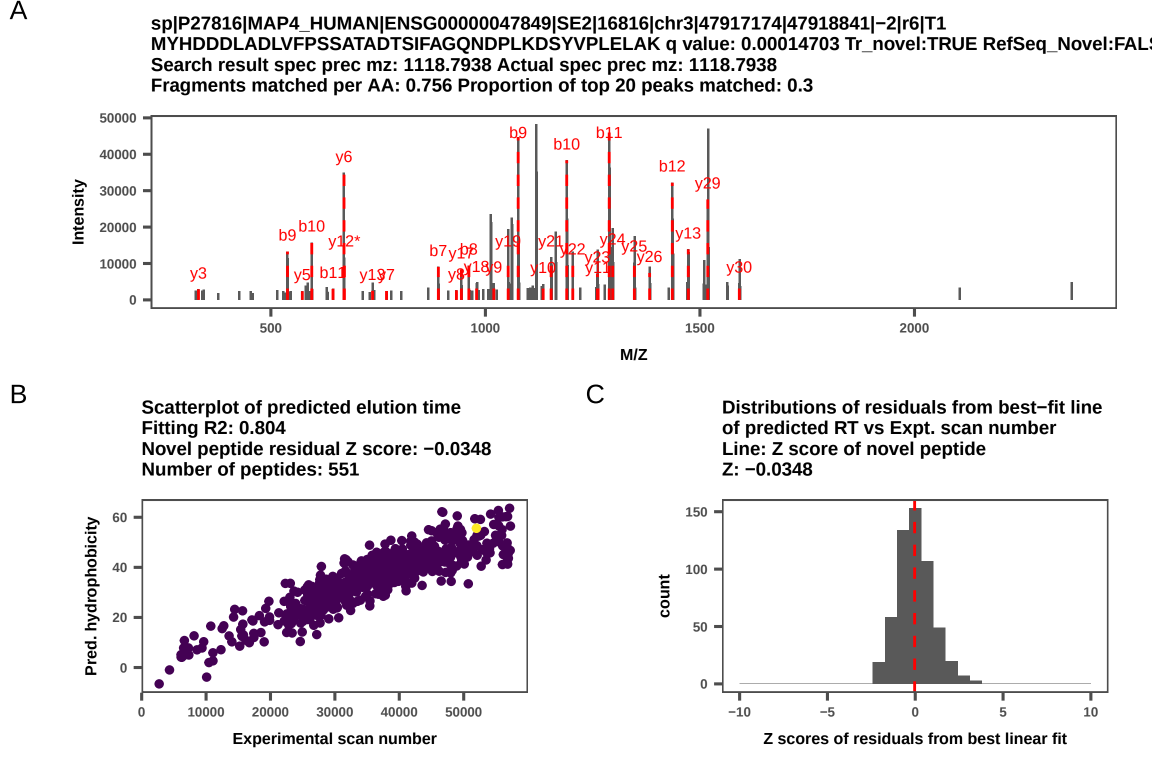
A
sp|P27816|MAP4_HUMAN|ENSG00000047849|SE2|16816|chr3|47917174|47918841|−2|r6|T1
MYHDDDLADLVFPSSATADTSIFAGQNDPLKDSYVPLELAK q value: 0.00014703 Tr_novel:TRUE RefSeq_Novel:FALS
Search result spec prec mz: 1118.7938 Actual spec prec mz: 1118.7938
Fragments matched per AA: 0.756 Proportion of top 20 peaks matched: 0.3
Intensity
M/Z
y3
b9
b10
y5
b11
y6
y12*
b7
b9
b10
b11
b12
y29
y13
y30
y21
y22
y23
y24
y25
y26
y10
y11
B
Scatterplot of predicted elution time
Fitting R2: 0.804
Novel peptide residual Z score: −0.0348
Number of peptides: 551
Pred. hydrophobicity
Experimental scan number
C
Distributions of residuals from best−fit line
of predicted RT vs Expt. scan number
Line: Z score of novel peptide
Z: −0.0348
count
Z scores of residuals from best linear fit
